# The Intentions of Migration Among Graduated and Postgraduate Sudanese Nursing Students 2022

**DOI:** 10.1155/nrp/5550685

**Published:** 2025-04-07

**Authors:** Shahenda Ateyat Allah Salih, Wafa Abdein Humza Bashir, Amal Mohammed Hamid, Amel Ahmed Hassan, Esraa Mohammed Alhussin, Maye Mohammed Merghani, Hammad Ali Fadlamola, Hawa Hamid, Warda Elshahat Hamed, Sitelbanat Osman Mohamed Ahmed, Ghada Abdelsalam Ahmed Eldeeb

**Affiliations:** ^1^College of Nursing, Jouf University, Sakaka, Saudi Arabia; ^2^Department of Nursing, College of Nursing and Health Sciences, Jazan University, Jazan, Saudi Arabia; ^3^Nursing Department, Prince Sultan Military College for Health Sciences, Dammam, Eastern, Saudi Arabia; ^4^Community Health Nursing, Alzaiem Al-Azhari University, Khartoum North, Khartoum, Sudan; ^5^Department of Nursing Education, King Fahad Hospital, Jeddah, Saudi Arabia; ^6^Medical Laboratory Program, Nahda College, Khartoum, Sudan; ^7^Department of Community Health Nursing, Nursing College, Taibah University, Madinah, Saudi Arabia; ^8^Psychiatric and Mental Health Nursing Department, Faculty of Nursing, Mansoura University, Mansoura, Egypt; ^9^Nursing Administration Department, Faculty of Nursing, Menoufiya University, Shibin Al Kawm, Egypt

**Keywords:** graduate, intention, migration, nurses, postgraduate, Sudan

## Abstract

**Background:** Migration of healthcare workers has become a foremost issue of health systems, generally from developing countries toward countries with higher income, producing destructive effects on health indicators.

**Aim:** This study was proposed to investigate the intentions of migration among graduated and postgraduate Sudanese nursing students and the causes behind their intentions to migrate and determine the proportions and characteristics (e.g., per gender and profession) of Sudanese nursing students, both graduated and postgraduate, who intend to migrate.

**Materials and Methods:** A descriptive cross-sectional survey was carried out in Khartoum State, Sudan, between January and April 2022. The study involved 321 Sudanese nurses, both graduate and postgraduate, who were selected through convenience sampling. Data collection was performed using a semistructured, self-administered questionnaire created by the researchers. The questionnaire, prepared in English, was distributed via Google Forms on social media platforms. The study received approval from the Research and Ethics Committee of Alzaiem Alazhari University. Informed consent was obtained from all participants prior to their involvement. Data analysis was conducted using the Statistical Package for Social Sciences (SPSS) software, Version 25. The results were presented in frequency tables and cross-tabulations. The Chi-square test was used to assess statistical significance, with a *p* value of < 0.05 considered significant.

**Results:** The current study reflected that half of the nurses (49.5%) were willing to migrate after graduation if provided the opportunity. More than a third (36.8%) of them are interested in migrating to the Gulf countries, while that quarter (24.9%) of them said their main reason for migration is to save money quickly. There was a significant association between five sociodemographic variables (age, sex, education program, marital status, and place of residence) and the reason for migration with a *p* value of 0.05.

**Conclusion:** The study concluded that there is high intention among graduated and postgraduate nursing students in Sudan to migrate and work outside Sudan.

## 1. Introduction

### 1.1. Background and Literature Review

The International Organization for Migration (IOM) defines immigration as the process of leaving one country with the intention of establishing residences in another [1]. The migration of healthcare workers has become a significant challenge for health systems, particularly as professionals move from developing countries to higher income nations. This trend has adverse effects on health indicators and is most prevalent in Sub-Saharan Africa and the Caribbean regions [2].

The presence of an adequate number of skilled and motivated healthcare workers is essential for the effective functioning of any health system. However, the international migration of workers, primarily from developing to developed countries, a phenomenon commonly referred to as “brain drain,” has become a growing concern. Brain drain occurs when a significant number of highly educated and skilled individuals leave their home country to work in nations offering better salaries and working conditions [3]. African countries have been identified as facing a severe shortage of healthcare workers. Despite shouldering 24% of the global disease burden, the continent represents only 2% of the global health workforce [4].

The quality, efficiency, and fairness of healthcare services rely heavily on the availability of a well-trained and skilled health workforce capable of meeting healthcare demands. The migration of health professionals significantly impacts the capacity of health systems. According to the IOM, around 20,000 African professionals from various fields leave the continent annually for industrialized Western countries in pursuit of personal and professional growth. Common drivers of international migration include economic challenges, institutional issues such as deteriorating healthcare systems, professional aspirations such as advancing qualifications, and political instability [5]. Several studies have highlighted both push and pull factors that drive the emigration of health professionals from Sub-Saharan Africa. Push factors include inadequate pay, poor working conditions, low job satisfaction, limited opportunities for further education or career advancement, an oppressive political environment, threats of violence, persecution of intellectuals, and the desire to secure better education and future prospects for one's children [6].

Nurses represent the largest professional group within the healthcare workforce, making up nearly 60% of all health professionals, including medical doctors, nursing personnel, midwives, dentists, and pharmacists [7]. Nurses, as key members of the healthcare team, are increasingly part of the global migration flow. Nursing is progressively becoming a mobile profession, with thousands of nurses—predominantly women—migrating annually in search of better pay, improved working conditions, career opportunities, professional growth, a higher quality of life, and personal safety [8]It is important not to ignore the role that job discontent with inadequate organizational and physical infrastructures plays in nurses' decisions to leave nursing, relocate, work in the private sector, or look for jobs with nongovernmental groups. Obstacles to fully utilizing specialist nursing expertise in healthcare settings, along with a lack of respect from doctors, also contribute to this issue [9]. The population of Sudan, an African nation that serves as a bridge between Middle Eastern and sub-Saharan African (SSA) nations, was estimated at 41,801,533 in 2018. The working-age population made up 78.8% of the total population. With 62.2% of the population under 25, the demographic profile is youthful due to the substantial natural population growth [10]. Only 5457 doctors, 12,106 nurses, and 17,343 midwives are employed in Sudan's public health system, according to a report released by the country's federal and state ministries of health. This is less than the WHO target of 4.45 doctors, nurses, and midwives per 1000 people, which is advised in the WHO health workforce requirements for universal health coverage [11]. In terms of the nation's nursing workforce, there is still a shortfall. To make up for this shortfall, the Sudanese government established the Academy of Health Sciences (AHS). There were five doctors for every nurse at the AHS when it was founded in 2006. The ratio shifted to 0.61 nurses per nurse in 2011 [12]. Other nations will gain from the money spent on nursing education. The issue of migration is not unique to Sudan. Since the 1960s, medical migration to Sudan has been recognized and has been increasing. The UK, Ireland, and the Gulf Area are the primary destinations for the nation's health professionals, primarily physicians. However, to a lesser extent, migration also affects nondoctor cadres. As nursing education shifts from vocational to university-level qualifications, removing the former market barrier, a new issue of high nurse migration is approaching. Trends of return migration and other types of foreign health professional immigration into the nation are countering this exodus. The balance, however, is still in favor of exodus with the country losing critical talent [13]. The number of skilled and semiskilled health workers, mainly physicians and nurses, who are leaving their country of origin, is steadily rising, especially in the past year. According to the most recent estimate, more than 60% of doctors have left the nation [14]. However, it was not stated how many nurses left Sudan to work abroad. The desire for higher wages and a higher standard of living may be the reason why health care personnel leave Sudan. Some people might also move for security, better training, education, and employment prospects. There is currently no single policy for tackling the outmigration issue, even with the recent efforts of the Ministry of Health and the Ministry of Finance to create a retention policy to lower the outmigration rate [15].

The conflict in Sudan began in April 2023 and has been going on for about two years. The conflict left the country in the worst humanitarian crisis its population has ever experienced, with famine threatening, people being displaced, and extreme hunger. Sudan's healthcare system has been decimated by the conflict, which has resulted in closures and disruptions, particularly in areas affected by the conflict, which make up more than two-thirds of the nation [16].

The economic and political instability in Sudan has a big impact on nursing students' migration plans. Nurses find it difficult to maintain a respectable standard of living due to economic issues such as low pay, rising unemployment, and inflation, which drives them to look for better chances outside. At the same time, unstable politics destroys the healthcare system, resulting in subpar working conditions, a shortage of financing, and security issues, all of which deter students from remaining. Their desire to leave is influenced by their peers' migration, their dread of an uncertain future, and their limited opportunities for professional advancement. Migration thus turns into a means of survival, providing better living conditions, job progression, and stability, but at the expense of making Sudan's healthcare workforce situation worse [17].

Over 10.4 million Sudanese, including university professors and researchers, were compelled to leave their country, which caused disruptions in the educational system. For instance, in Khartoum State, where there are 73% nongovernmental and 39% public universities, the rapid support forces have taken over all of them. As a result, the universities have been forced to close, and students have started to look for costlier or nonexistent options for continuing their education [18].

This study was designed to explore the intentions of migration among graduated and postgraduate Sudanese nursing students and the causes behind their intentions to migrate and to determine the proportions and characteristics (e.g., per gender and profession) of Sudanese nursing students both graduated and postgraduate who intend to migrate.

## 2. Materials and Methods

A descriptive cross-sectional study was conducted at Khartoum State in Sudan during a period extended from January to April 2022. The study population were graduated and postgraduate nursing students (BSc, MSc, and PhD) studied at different governmental and private Sudanese universities, who were willing to participate in the study.

### 2.1. Sampling Technique and Sample Size

The sample size was calculated by using the following formula:

(1) $$\begin{array}{c} n=p\left(1-p\right)\;{\left(z/e\right)}^{2}\;n=0.5\;\left(1-0.5\right)\;{\left(z/e\right)}^{2}=0.5\;\left(0.5\right)\;{\left(1.96/0.05\right)}^{2}=384.2=385. \end{array}$$

Calculations showed that the sample size was 385, with 321 (83%) nurses. Convenience sampling was used to pick the students that replied. Because student selection was performed outside of class hours and with each student's consent, convenience sampling was an appropriate sample technique. All participants gave their agreement prior to their participation in the study, which was approved by Alzaiem Alazhari University's Research and Ethics Committee. The study was optional, and participants were guaranteed that those who chose not to participate or who opted out after providing consent would not be victimized.

### 2.2. Data Collection Method and Tools

The study tool was a pretested, semistructured, self-administered questionnaire that the researchers created using a Google Form and shared on social media in English, the language of education at Sudanese nursing faculties. Students' basic demographic data, including age, gender, educational program, residence, and marital status, were gathered in the first section of the questionnaire. In the second section, students were asked to list their reasons for wanting to migrate, as well as their short-term (further education or training or temporary work) and long-term (stable job and life) migration destinations. Both “home country” and “a foreign country” were options for the migratory intentions; if they selected the latter, they had to put down the name of the country.

### 2.3. Data Analysis

Data were analyzed by Statistical Package for Social Sciences (SPSS) version 25, and the results were displayed in frequency tables and cross tabulations. A chi-square test of statistical significance was used in the analysis, and the level of statistical significance was determined by a *p* value of < 0.05.

### 2.4. Ethical Consideration

This study received the ethical approval from the local board of the Faculty of Medical Technical Sciences at Alzaiem Alazhari University.

## 3. Results

The study conducted among 321 graduated and postgraduate nursing students in Sudan provides valuable insights into their migration intentions and the influence of various sociodemographic characteristics.  Table 1 shows that the majority (50.8%) of the participants fall within the 18–24 years, followed by smaller proportions in the 31–36 years (17.4%), 37–42 years (12.1%), and over 42 years (9.3%) categories, with 10.3% unspecified. Gender distribution shows a predominance of females (81.3%) compared to males (18.7%). Marital status indicates that most participants are single (61.1%), while 36.1% were married, with very few divorced (1.9%) and widowed (0.9%). Educational attainment reveals that 67.6% are pursuing B.Sc., while 16.5% and 15.9% are enrolled in M.Sc. and Ph.D. programs, respectively; the majority reside in urban areas (76.3%), with a smaller fraction living in rural settings (23.7%). This finding aligns with Aaron's results, which highlighted that most respondents were young adults and the majority were single. Additionally, a significant portion of the respondents were from urban areas [6].

The distribution of specialties or areas of experience among the respondents is as follows: General nursing is the most prominent specialty, comprising 32.1% of the total, followed by medical–surgical nursing at 25.2%, pediatric nursing accounts for 6.5%, and obstetrics and gynecological nursing for 5.6%. Other specialties, such as surgical nursing, community health nursing, and psychiatric nursing, each represent 5.3%, while nursing education accounts for 3.7%. Smaller proportions include anesthetic nurses (2.2%), cardiothoracic nurses (0.6%), emergency nurses (1.2%), oncology nurses (0.9%), and ICU nurses (1.6%). A minor percentage (1.9%) falls under “other” categories.

Regarding respondents' willingness to migrate after graduation and their preferred destinations, a majority (49.5%) expressed that they would migrate from Sudan if given the opportunity, while 35.2% are currently considering migration and 15.3% are not willing to migrate. Among those interested in migration, the Gulf countries are the most preferred destination (36.8%), followed by Europe (20.6%) and America (18.1%). England is a destination for 6.9% of respondents, while smaller proportions prefer Australia (1.6%) or other unspecified countries (0.9%). 15.3% are unwilling to migrate account for the remaining group.

Table 2 presents the distribution of reasons for migration and plans for returning back to Sudan among 321 respondents. The main reasons for migration include the desire to save money quickly for a purpose (24.9%), improving quality of life (16.5%), and gaining experience or improving skills abroad (9.3%). Other reasons include seeking specialist training (8.7%), better working and career opportunities (2.5%), and personal or family-related reasons (5.3%). A small percentage cited insufficient opportunities for promotion in Sudan (0.9%) or the belief that there is no future in the country (2.8%). Regarding their plans for returning to Sudan, 26.5% of respondents plan to work for less than 5 years before returning, while 17.1% aim to work between five and 10 years. A smaller proportion (10.3%) intends to return immediately after training, while 20.9% do not plan to return at all. The remaining respondents (15.3%) are not willing to migrate.

Table 3 reveals several key differences and similarities across various demographic groups, including age, gender, marital status, education level, and place of residence.

The study on migration intentions of Sudanese nursing students revealed several notable trends and differences across various sociodemographic factors. A significant proportion of participants (49.5%) expressed a willingness to migrate, with migration intentions showing distinct patterns across different age groups. Among those aged 18–24, 25.2% expressed a desire to migrate primarily to save money quickly. In the 31-36 age group, 30.8% were motivated by the desire to improve their quality of life, while 30% of respondents in the 37–42 age group were inclined to pursue further training abroad. Statistical analysis revealed that age was significantly associated with migration reasons (*p* < 0.05), highlighting that those motivations varied considerably with age.

Gender analysis revealed that the sample consisted predominantly of females (81.3%), with males comprising 18.7% of the sample. While both genders were primarily motivated by financial reasons for migration (24.9%), females showed a slightly higher inclination toward improving their quality of life (16.5%) compared to males (16.7%). However, no significant relationship was found between gender and reasons for migration (*p* > 0.05), suggesting that gender did not substantially affect migration intentions.

Regarding marital status, the study found that 61.1% of participants were single, with a higher propensity to migrate among this group (24%) driven by the desire to save money quickly. Married individuals, in contrast, were more likely to cite family-related reasons for migration (5.3%). A significant association was observed between marital status and migration reasons (*p* < 0.05), indicating that marital status influenced migration motivations, particularly in relation to family considerations.

The education level of participants revealed that 67.6% held a B.Sc., 16.5% a Master's degree, and 15.9% a PhD. B.Sc. graduates were predominantly motivated by financial reasons (23.5%), while M.Sc. and PhD holders were more focused on specialist training and further education (7.5% and 5.9%, respectively). A significant relationship was found between the education level and reasons for migration (*p* < 0.05), suggesting that higher educational qualifications are associated with motivations that go beyond immediate financial gain, such as skill development and specialized training.

In terms of place of residence, 76.3% of participants lived in urban areas. Urban residents were more likely to migrate for financial reasons (27.8%) compared to rural residents (15.8%). However, no significant relationship was found between the place of residence and reasons for migration (*p* > 0.05), indicating that while there were some differences in the motivations for migration between urban and rural respondents, this difference was not statistically significant.

Overall, the study underscores several key insights into the migration intentions of Sudanese nursing students. Financial motivations, particularly the desire to save money quickly, were the most common reasons for migration across all demographic groups. Younger individuals, particularly those aged 18-24, were more focused on immediate financial gains, while older groups were more likely to prioritize improving their quality of life. The education level also played a significant role, with higher education correlating with motivations for specialist training rather than immediate economic benefits. These findings highlight the complex and varied nature of migration intentions, with demographic factors influencing the reasons for migration in distinct ways.

## 4. Discussion

The number of international migrants' nurses on the move every year continues to increase mainly after the economic collapse that country is experiencing, which opens the door for the emigration of all those who aspire to reform their economic conditions.

This study is designed to explore the migration intentions of graduate and postgraduate Sudanese nursing students and the reasons behind their migration intentions and determine the proportion and characteristics of nursing students. The total number of 321 graduated and postgraduate nurses was enrolled in this study; two-thirds of them, 67.6%, have a BSc degree; the majority (81.3%) were female, 61.1% were single, 50.8% were in the age group between 18 and 24 years old, and 76.3% were from urban areas. This result is consistent with the result by Aaron, who said that respondents with ages between 18 and 25 years were in the majority (75%) of students who were single and constituted 87% of respondents. While 76.3% are from urban areas, this result is inconsistent with the same study, who said rural posting would not influence their intention to migrate were in the majority (77%) [6]. Regarding the nursing specialization of the participants, 32.1% were general nurses, while 25.2% were specialized in medical–surgical nursing. This finding is consistent with a study by Smith et al., which reported that 32.7% of nurses in their sample were in medical–surgical specialties [19]. Similarly, the Gohs and Lopez study also found that 32.7% of nurses were medical–surgical nurses, further supporting these findings [20].

The high migration intentions among Sudanese nursing students were notable, with 84.7% indicating that they were either currently considering migration or were willing to migrate if given the opportunity. Of those, 35.2% were actively considering migration, and 49.5% would migrate if the opportunity arose. The study also identified the most popular destinations for migration, with the Gulf countries, particularly Saudi Arabia, being the top choice (36.8%), followed by Europe (20.6%) and the United States (18.1%). These findings align with Mohammed et al., who also reported that the majority of medical students from Sudan aimed to migrate to the Gulf countries, with 29% of participants choosing Saudi Arabia and others opting for destinations such as the UAE, the UK, and the USA [14].

Globally, the shortage of a well-trained healthcare workforce has reached critical levels, and this shortage is exacerbated by the migration of skilled professionals to more developed countries. For instance, in Ghana, it is estimated that over 56% of doctors and 24% of nurses trained in the country are working abroad, reflecting a significant brain drain in the healthcare sector [6]. This study on Sudanese nurses reveals a similar trend, with migration being primarily motivated by several factors. The top reasons for migration identified in this study include the desire to save money quickly, seek further specialization and training, access better equipment and facilities, improve the quality of life, and pursue personal reasons. These motivations align closely with findings from previous research, where financial incentives, opportunities for career advancement, and the pursuit of a better quality of life were commonly cited as driving forces behind the migration of healthcare workers [21].

Interestingly, the study found that while the reasons for migration varied slightly across different groups, the common thread among most participants was the pursuit of better financial conditions and professional development opportunities. This trend is consistent with global patterns observed in other regions, where economic and professional motivations are often the primary drivers behind healthcare workers' decisions to move abroad [22]. Moreover, the study found significant associations between various sociodemographic factors, including age, gender, marital status, educational level, and place of residence, and the reasons for migration. Specifically, younger age groups and those with higher educational levels were more likely to migrate for further training and career progression, while financial motivations were more prominent among those with lower educational levels.

These findings corroborate the model proposed by Toyin et al., which identified key predictors of migration such as age, marital status, rural posting, the presence of relatives abroad, work opportunities without relatives, the availability of higher salaries, career advancement prospects, further education, and children's education [21]. In this study, a significant association was found between five sociodemographic variables—age, gender, marital status, education level, and place of residence—and migration intentions. The results emphasize the multifaceted nature of migration decisions, influenced by a combination of personal aspirations, professional goals, and socioeconomic factors.

Understanding these underlying factors is essential for policymakers and stakeholders in healthcare to address the challenges posed by the migration of healthcare workers. It is crucial to develop strategies that not only encourage the retention of skilled professionals within their home countries but also enhance the working conditions, career development opportunities, and financial incentives available to them. By tackling the root causes of migration, countries can better safeguard their healthcare workforce and ensure that skilled professionals remain committed to improving healthcare standards within their local contexts.

## 5. Conclusion

The study found that Sudanese nursing students, primarily female, young, and single, are highly motivated to migrate and work outside Sudan, with financial rewards being the primary reason. Nearly a third plan to return within 5 years, highlighting the need for comprehensive policies to manage outmigration dynamics among the health workforce.

## 6. Recommendations

The recommendations that emerged from this study results and discussions for the health policy makers, stakeholders, and all health system partners are to develop and implement comprehensive retention strategies and policies, like what it is an important mechanism to reduce health workers out migration implication on the health system.

• Improve the graduate nursing rewards because it was the first pushing cause (poor financial rewards in Sudan) and also the first pulling factor (good rewards in other recipient countries) to migration by improving the salary packages.
• Strengthen the health management system.

## 7. Limitations

While the study provides valuable insights into migration intentions, there are limitations that should be considered, including restricted sample representativeness due to the online survey method and its focus on Khartoum State, which may not capture the diversity of Sudanese nursing students. Convenience sampling further reduces generalizability. Future research should expand the sample size and geographic scope while incorporating qualitative methods to explore personal and emotional factors influencing migration.

## Figures and Tables

**Table 1:** Summary of sociodemographic characteristics, migration intentions, and preferred destinations.

Category	Subcategory/item	Frequency	Percentage (%)
Sociodemographic data	*Age*		
	18–24 years	163	50.8
	31–36 years	56	17.4
	37–42 years	39	12.1
	More than 42 years	30	9.3
	*Gender*		
	Male	60	18.7
	Female	261	81.3
	*Marital status*		
	Single	196	61.1
	Married	116	36.1
	Divorced	6	1.9
	Widowed	3	0.9
	*Education program*		
	B.Sc.	217	67.6
	M.Sc.	53	16.5
	PhD	51	15.9
	*Place of residence*		
	Urban	245	76.3
	Rural	76	23.7

Nursing specialty	General nurses	103	32.1
	Medical/surgical nurses	81	25.2
	Other nursing specialties	137	42.7

Willingness to migrate after graduation	*Migration intent*		
	Currently considering migration from Sudan	113	35.2
	Would migrate from Sudan, if provided the opportunity	159	49.5
	Not willing to migrate from Sudan	49	15.3
	*Preferred destinations*		
	Gulf countries	118	36.8
	America	58	18.1
	Europe	66	20.6
	England	22	6.9
	Australia	5	1.6
	Other	3	0.9
	Not willing to migrate	49	15.3

*Note: n* = 321.

**Table 2:** Distribution of reasons for migration and return plans.

Items	Frequency (*n* = 321)	Percent (%)
*Reasons for migration*		
Want to save money quickly for a purpose	80	24.9
For specialist and further training	28	8.7
Good equipment/facilities	19	5.9
Improve quality of life	53	16.5
Improved skills/to gain experience abroad	30	9.3
Better working/career opportunities	8	2.5
Personal reason	16	5.0
Family-related reasons	17	5.3
Insufficient opportunities for promotion in Sudan	3	0.9
There is no future in Sudan	9	2.8
To travel and see the world	2	0.6
No specific reason	7	2.2
I am not willing to migrate from Sudan	49	15.3
Total	**321**	**100**

*Return plan to Sudan (if willing to migrate)*		
Immediately after training	33	10.3
Work < 5 years and return	85	26.5
Work 5–10 years and return	55	17.1
Work > 10 years and return	32	10.0
Never return back	67	20.9
I am not willing to migrate	49	15.3
Total	**321**	**100**

*Note: n* = 321.

**Table 3:** Association between reasons for migration and sociodemographic characteristics.

Reason for migration	Associated factor	Key observations	*p* value	Research implications
Want to save money quickly for a purpose	Age	Significantly higher among 18–24 years (25.2%) and 37–42 years (30.0%).	0.022⁣^∗^	Indicates financial motivation is strongest in early and mid-career individuals, relevant for the policy targeting these age groups.
Want to save money quickly for a purpose	Education level	Higher prevalence among PhD holders (29.4%) and Master's degree holders (26.4%).	0.039⁣^∗^	Suggests financial security as a key driver for migration among highly educated individuals.
Want to save money quickly for a purpose	Gender	Similar proportions among males (26.7%) and females (24.5%).	0.751	Gender does not significantly influence financial motivations for migration.
Want to save money quickly for a purpose	Residence	More common in urban residents (27.8%) than rural residents (15.8%).	0.727	No strong relationship, but urban respondents show slightly greater interest, possibly due to better opportunities abroad.
Want to save money quickly for a purpose	Marital status	Highest among widowed respondents (66.7%).	0.385	Highlights unique motivations of widowed individuals, though not statistically significant.
Improve quality of life	Age	Most prevalent among 31–36 years (30.8%) and 37–42 years (23.3%).	Not significant	Middle-aged individuals are more likely to seek migration to improve life quality; useful for targeted programs.
Improve quality of life	Education level	Higher among PhD holders (25.5%) and Master's degree holders (22.6%).	Not significant	Advanced degree holders prioritize better living conditions, though not statistically conclusive.
Improve quality of life	Residence	Higher among rural respondents (19.7%) compared to urban respondents (15.5%).	Not significant	Suggests that rural individuals view migration as a pathway to life improvement, relevant for rural development strategies.
Gain skills or experience abroad	Age	Most prevalent among 18–24 years (12.9%).	Not significant	Indicates younger individuals see migration as an opportunity to enhance professional skills early in their career.
I am not willing to migrate	Age	Resistance to migration is higher among 37–42 years (30.0%) and > 42 years (24.2%).	Not significant	Older individuals are less likely to migrate, suggesting rootedness or lower adaptability to migration.

*Note: n* = 321.

⁣^∗^Statistically significant findings (*p* value < 0.05).

## Data Availability

The data supporting the findings of this study and any additional datasets generated and analyzed during this study are available with the corresponding author upon reasonable request.

## References

[1] Geiger M., Koch M. (2018). World Organizations in Migration Politics: The International Organization for Migration. *Journal of International Organization Studies*.

[2] Anduaga-Beramendi A., Beas R., Maticorena-Quevedo J., Mayta-Tristán P. (2019). Association between Burnout and Intention to Emigrate in Peruvian Health-Care Workers. *Safety and Health at Work*.

[3] Kamarulzaman A., Ramnarayan K., Mocumbi A. O. (2022). Plugging the Medical Brain Drain. *The Lancet*.

[4] WHO (2023). *The World Health Report: 2006: Working Together for Health*.

[5] Kallio K. P., Häkli J. (2023). Trapped in (In) Visibility: Contested Intercorporeality in Undocumented Migrants’ Lives. *Geopolitics*.

[6] Showers F. (2023). I Just Ended up in Nursing: Inequalities in Health Professions Education and Migration Aspirations Among Medical and Nursing Students in Ghana. *SSM-Qualitative Research in Health*.

[7] Kingma M. (2018). *Nurses on the Move: Migration and the Global Health Care Economy*.

[8] Who (2020). State of the World’s Nursing 2020: Investing in Education, Jobs and Leadership.

[9] Who (2017). *Sudan Health Profile 2015*.

[10] Sudan J. I. N. FORCED MIGRATION, INTERNAL DISPLACEMENT AND.

[11] AbuAgla A., Yousif N., Badr E. (2013). *Understanding the Labour Market of Human Resources for Health in Sudan*.

[12] Badr E. (2011). Migration of Health Professionals in Sudan: Need for a National Policy. *Sudanese J Public Health*.

[13] Taha F., Bjørkelo A. (2016). *The Crisis of Higher Education in Sudan with Special Reference to the University of Khartoum, 1956–2014*.

[14] Mohamed E. Y., Balla S., Abdulaal A., Albary M. A., Mukhtar A. M. (2015). Postgraduation Migration Intentions of Medical Students in the Faculty of Medicine, University of Khartoum. *World Journal of Pharmacy and Pharmaceutical Sciences*.

[15] Elamin A., Abdullah S., ElAbbadi A. (2024). Sudan: from a Forgotten War to an Abandoned Healthcare System. *BMJ Global Health*.

[16] Badri R., Dawood I. (2024). The Implications of the Sudan War on Healthcare Workers and Facilities: a Health System Tragedy. *Conflict and Health*.

[17] Çinar H. Y., Özer A. (2023). Internal and External Factors behind the Instability in Sudan. *Perceptions: Journal of International Affairs*.

[18] Alamin N. K. N., Idris A. A. A., Khugali G., Kalcon G. O. A., Khaleel M. (2024). Science in Times of War: Reflections from Sudan. *ASFI Research Journal*.

[19] Smith J. B., Herinek D., Woodward-Kron R., Ewers M. (2022). Nurse Migration in Australia, Germany, and the UK: A Rapid Evidence Assessment of Empirical Research Involving Migrant Nurses. *Policy, Politics, & Nursing Practice*.

[20] Goh Y., Lopez V. (2016). Job Satisfaction, Work Environment and Intention to Leave Among Migrant Nurses Working in a Publicly Funded Tertiary Hospital. *Journal of Nursing Management*.

[21] Toyin-Thomas P., Ikhurionan P., Omoyibo E. E. (2023). Drivers of Health Workers’ Migration, Intention to Migrate and Non-migration from Low/middle-Income Countries, 1970–2022: A Systematic Review. *BMJ Global Health*.

[22] Hajian S., Yazdani S., Jadidfard M. P., Khoshnevisan M. H. (2020). Factors Influencing the Migration Intention of Health Professionals in Low-And-Middle Income Countries: Critical Review with a Theoretical Model. *Journal of Contemporary Medical Sciences*.

